# Technical Aspects and Validation of a New Biofeedback System for Measuring Lower Limb Loading in the Dynamic Situation

**DOI:** 10.3390/s17030658

**Published:** 2017-03-22

**Authors:** Marco Raaben, Herman R. Holtslag, Robin Augustine, Rutger O. van Merkerk, Bart F. J. M. Koopman, Taco J. Blokhuis

**Affiliations:** 1 Department of Surgery, University Medical Center Utrecht, Heidelberglaan 100, 3584 CX Utrecht, The Netherlands; 2 Department of Rehabilitation Medicine, Academic Medical Center Amsterdam, Meibergdreef 9, 1105 AZ Amsterdam, The Netherlands; h.r.holtslag@amc.uva.nl; 3 Department of Engineering Sciences, Uppsala University, Lägerhyddsvägen 1, Box 534, 751 21 Uppsala, Sweden; Robin.Augustine@angstrom.uu.se; 4 Pontes Medical, University Medical Center Utrecht, Heidelberglaan 100, 3584 CX Utrecht, The Netherlands; r.vanmerkerk@prolira.com; 5 Department of Biomechanical Engineering, University of Twente, Drienerlolaan 5, 7522 NB Enschede, The Netherlands; h.f.j.m.koopman@utwente.nl; 6 Department of Surgery, Maastricht University Medical Center+, P. Debyelaan 25, 6229 HX Maastricht, The Netherlands; taco.blokhuis@mumc.nl

**Keywords:** biofeedback, ambulatory monitoring, lower limb loading, weight-bearing

## Abstract

Background: A variety of techniques for measuring lower limb loading exists, each with their own limitations. A new ambulatory biofeedback system was developed to overcome these limitations. In this study, we described the technical aspects and validated the accuracy of this system. Methods: A bench press was used to validate the system in the static situation. Ten healthy volunteers were measured by the new biofeedback system and a dual-belt instrumented treadmill to validate the system in the dynamic situation. Results: Bench press results showed that the sensor accurately measured peak loads up to 1000 N in the static situation. In the healthy volunteers, the load curves measured by the biofeedback system were similar to the treadmill. However, the peak loads and loading rates were lower in the biofeedback system in all participants at all speeds. Conclusions: Advanced sensor technologies used in the new biofeedback system resulted in highly accurate measurements in the static situation. The position of the sensor and the design of the biofeedback system should be optimized to improve results in the dynamic situation.

## 1. Introduction

Accurate measurements of lower limb loading is relevant in many fields, such as lower limb injuries, diabetes mellitus, stroke, footwear design, sport biomechanics, and injury prevention [1]. Systems for monitoring lower limb loading have therefore become a growing field of interest since the first introduction of such a system in 1974 [1,2]. Current load monitoring systems can be classified in platforms and (ambulatory) biofeedback devices [2]. 

Force platforms are accurate and easy to use for load measurements in static and dynamic situations [2]. Force platforms placed inside a treadmill are even more beneficial as walking speed can be controlled. Moreover, loading can be controlled by the use of overhead body weight-support systems. The biomechanics of treadmill walking, however, differs from overground walking [3]. Healthy individuals have shown modified muscle activation patterns and thereby modified joint movements and forces during treadmill walking [4]. In addition, greater cadence, smaller stride length and stride time, as well as different ground reaction forces were found in the elderly [5]. Although some ambulatory platforms exist, most platforms are restricted to research laboratories, which prevents clinical implementation [1,2]. 

The development of biofeedback systems seems promising as lower limb loading can be measured in the dynamic situation. A variety of (semi) portable ambulatory devices are currently available. Although devices such as the F-Scan (Tekscan Inc., Boston, MA, USA), Pedar^®^ Force Monitoring System (Novelgmbh, Munich, Germany), GaitShoe (Massachusetts Institute of Technology Media Laboratory, Cambridge, MA, USA) and SmartStep (Andante Medical Devices, Beer Sheva, Israel) have been shown to obtain accurate results [6,7,8,9,10], important limitations still remain. These limitations include the use of cables, sensor migration, creep, temperature drift, humidity drift, hysteresis, limited storage capacity, and a lack of commercial availability [2,11]. Moreover, ambulatory visual feedback about lower limb loading during gait is not possible with any of these systems. At most, an auditory warning signal or postresponse feedback can be given after a training exercise, and this limits the performance and effectiveness for clinical use [12,13]. These drawbacks together with high prices prevent worldwide clinical implementation of these devices.

To overcome limitations of previous devices, our research group developed a new ambulatory biofeedback system for measuring lower limb loading in the dynamic situation (SensiStep, Evalan BV, Amsterdam, The Netherlands). Important aspects in the development were usability and accuracy for clinical application, as well as the opportunity to provide ambulatory visual feedback in real-time. We aimed to meet the requirements as previously described by Razak et al. [1], including a high level of mobility, limited cabling, correct sensor placement, low power consumption, and low cost. In addition, the system should be able to continuously monitor patients and provide accurate and real-time feedback about lower limb loading to both patient and healthcare professional. 

The aim of this first study is to (1) describe the technical aspects of the system and (2) validate the accuracy of the data generated by the system in both static and dynamic situations. Bench press testing was used to validate the system in the static situation. The obtained force measurements in the dynamic situation were compared to a dual-belt instrumented treadmill (R-Mill, ForceLink, Culemborg, The Netherlands). 

## 2. Materials and Methods 

### 2.1. Technical Aspects of the System

#### 2.1.1. Set-Up of the System

**Figure 1:**
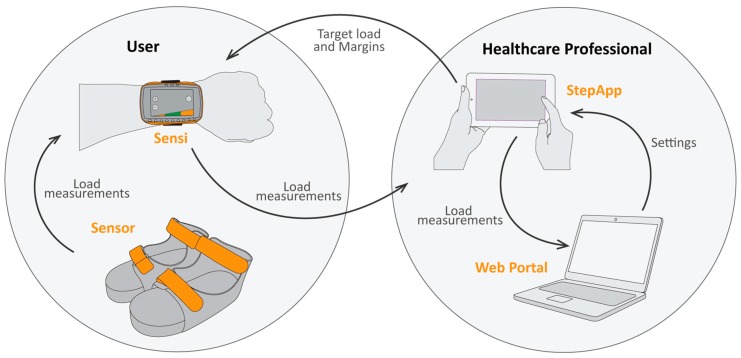
Schematic representation of the biofeedback system with the five components: the insole sensor sends load measurements wireless and in real-time to a wrist device (Sensi), which acts as feedback instrument for the patient. These load measurements are then sent wireless and in real-time to a tablet, which acts as feedback instrument for the healthcare professional. The healthcare professional sets the desired target load and margins in the tablet (StepApp). Finally, all sessions are saved on a secured Web Portal.

The biofeedback system consists of five different components as shown in Figure 1: (1) a force sensor; (2) custom-made sandals; (3) a wrist device; (4) a tablet; and (5) a secured Web Portal. The force sensor (dim: 55 × 75 × 14.9 mm, weight: 190 g) is placed inside a specific position in the sole of the custom-made sandal. This ensures the sensor is always positioned correctly under the midfoot of the patient. The in-sole sensor sends load measurements wireless and in real-time via Bluetooth 4.0 to a wrist device. This wrist device acts both as data aggregator as well as feedback instrument for the patient. Real-time feedback about lower limb loading is given by the wrist device via green and red light emitting diodes (LEDs). Simultaneously, this device sends the load measurements wireless and in real-time via Bluetooth to a tablet (ASUS Tablet, Model: K01A, ASUSTek COMPUTER INC., Taipei, Taiwan). On this tablet, a specially developed application is installed. This application can be used by the healthcare professional to set the target load, including a range or threshold around this target. During gait, the tablet shows real-time graphic illustrations of all steps, including the predetermined target load. All measurements are saved on a secured Web Portal for postresponse feedback. All electronic components contain rechargeable batteries. The components of the system will be discussed in detail in the following sections.

#### 2.1.2. The Sensor

**Figure 2:**
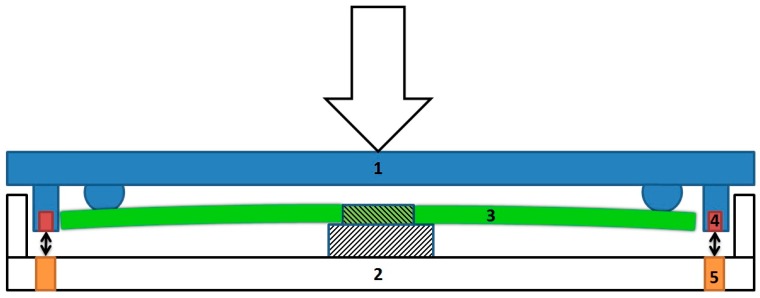
Schematic representation of the sensor. The sensor consists of six different parts: (1) the cover and (2) bottom plate consist of high quality non-ferromagnetic aluminum 7075 (AlZn5,5MgCu), (3) the laser-cut flat spring is made of strong and non-corrosive stainless steel (RVS AISI 301), (4) the N45 magnets are made of NdFeB (Neodymium–Iron–Boron), (5) the Hall sensors (Honeywell SS49E), and (6) a U3-HV USB data acquisition module (LabJack, Lakewood, CO, USA, not shown in figure). A force exerted on the cover plate (1) will be transferred via the beads to the four flat springs (3). Depending on the applied force, these springs will bend more or less, which results in movement of the magnets (4) and thus a change in the magnetic field at each of the Hall sensors (5). This creates changes in the output signal of the Hall sensors, which makes it possible to calculate the total force exerted on the spring by using the correct signal processing and algorithm.

The sensor consists of six different parts as shown in Figure 2. The cover plate (1) is connected via small beads to four flat springs (3), which are supported in the center by the bottom plate (2). Magnets (4) and Hall sensors (5) are placed at each corner. Each of these Hall sensors measure the magnetic field created by one of the magnets. A force exerted on the cover plate will be transferred via the beads to the four flat springs. Depending on the applied force, these springs will bend more or less, which results in movement of the corners and thus a change in the magnetic field at each of the Hall sensors. This creates changes in the output signal of the Hall sensors, which makes it possible to calculate the total force exerted on the spring by using the correct signal processing and algorithm. The springs have been designed to behave linearly according to the formula B=k∗δ, where B is the force exerted on the spring, k is the constant of the spring, and δ equals the deflection of the spring. Experimental and simulation models have shown that the materials used in the design of the sensor ensure that the bending load is measured (data not shown). By using this sensor, it is possible to measure the axial force exerted on the lower extremities in the dynamic situation. 

The sensor must be calibrated once after manufacturing by applying a constantly increasing force between 0 and 2000 N (incremental steps of 100 N) in the middle of the sensor. Subsequently, data from all four sensors should be recorded independently. For each sensor, a third degree polynomial function can be fitted according to the gathered data and used to calculate the force per sensor. The total force is simply the sum of all four individual sensors. After successful calibration, the sensor can be used without recalibration by the healthcare professional or patient. 

#### 2.1.3. The Sandals

The biofeedback system contains custom-made sandals (size 37–40–43–45, range: 35–47). The sensor fits perfectly in the sole of these sandals. This ensures easy and correct sensor placement by the treating physician or patient. The sandals are designed to allow normal gait patterns. 

#### 2.1.4. The Wrist Device

The in-sole sensor sends its load measurements via Bluetooth 4.0 (Bluetooth Low Energy, BLE) to the data aggregator, which is a wrist device worn by the patient. BLE is chosen because it is wireless, has low energy consumption, and has expanding possibilities in the future. The wrist device contains two-color LEDs (red/green) that can be programmed to provide real-time feedback about lower limb loading to the patient. The threshold that is set in the tablet (see below) corresponds to these LEDs. Green light means correct loading, and red light means loading below or above the threshold. The wrist device continuously sends data via Bluetooth to the tablet if the two devices are connected. 

#### 2.1.5. The Tablet

The healthcare professional sets the desired target load, including a range around this target, in the application of the tablet. This target load, including range, is shown as a green bar on the tablet screen. Steps are displayed in real-time on the tablet, providing insight in lower limb loading during exercises to both the patient and the healthcare professional. The step detection in the application is a mathematic parameter based on dynamic loading; when a load of 10% of the target load is measured, a start point is set. If the patient now exceeds at least 20% of the given target load and lessens the load to below the 10% line within a time frame of 0.2 to 2.0 s from the start point, it is registered as a step. For postresponse feedback and/or research purposes, all data are sent via Wi-Fi to a secured Web Portal. 

### 2.2. Validation of the Biofeedback System

#### 2.2.1. Static Loading

**Figure 3:**
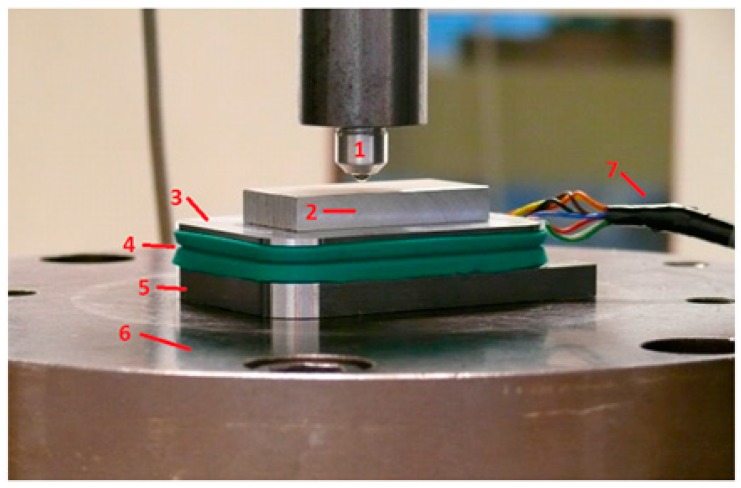
Validation of the sensor in the static situation using a bench press. The sensor (3–5) was placed between a solid underground (6) and a solid plate (2) to increase the area of applied force by the bench press (1). Incremental forces from 0 to 1000 N were applied to the sensor. For this experimental set-up a cable (7) was connected from the sensor to a laptop to analyze the data in the static situation.

Sensor accuracy was tested in the static situation using a bench press. The experimental design is illustrated in Figure 3. An increasing force ranging from 0 to 1000 N was applied by the bench press, and the sensor outcome was measured. 

#### 2.2.2. Dynamic Loading

The biofeedback system was also tested in the dynamic situation, and obtained results were compared to a dual-belt instrumented treadmill (R-Mill, ForceLink, Culemborg, The Netherlands). Ten healthy volunteers with unrestricted mobility were equipped with the biofeedback system, consisting of the custom-made sandals, the force sensor, and the wrist device. The tablet was held by the investigator. The participants walked with the biofeedback system on the R-Mill at different speeds: 0.5, 1.0, 2.0, 3.0, and 4.0 km/h for 90 s at each speed. All participants gave their informed consent, and the study protocol was approved by the local ethics committee of the University Medical Center Utrecht.

Parameters of interest were peak load and loading rate, as these parameters were considered to be most relevant for this first validation study. Especially the loading rate, which is defined as the steepness of the loading curve, has shown clear correlations with length of stay in the nursing home in a previous study using the FeetB@ck system [14]. Specific Matlab (Matlab R2014a, MathWorks) routines were developed to convert the raw data to parameters of interest. Average peak loads and loading rates were calculated per participant per speed by taking the average of all single steps at that specific speed. The absolute and relative error were then calculated according to the formulas E(abs)=Fb−Fr and $E\left(rel\right)=\frac{E\left(abs\right)}{Fr}$, respectively, where *E*(*abs*) and *E*(*rel*) are the absolute and relative error, Fr the force measured by the R-mill and Fb the force measured by the biofeedback system.

## 3. Results

**Figure 4:**
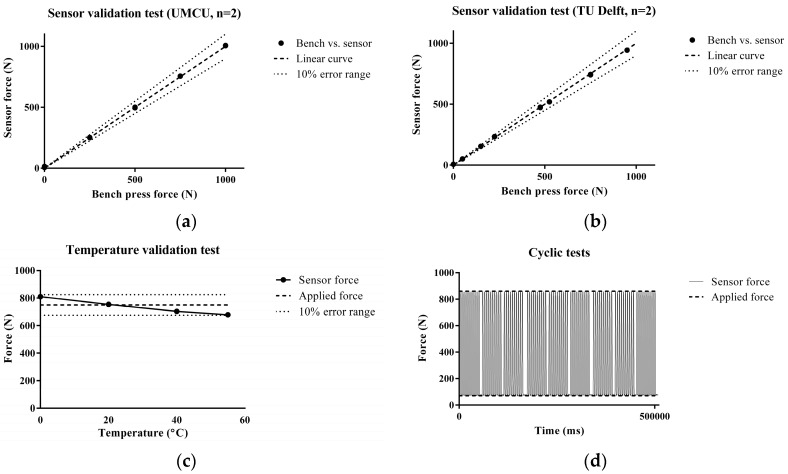
Validation of the sensor in the static situation. (**a,b**) Tests at the University Medical Center Utrecht (n = 2) and Technical University Delft (n = 2). The black dots indicate the applied force by the bench press versus the measured force by the sensor; (**c**) Sensor validation test at a broad range of temperatures (0–55 °C) at constant force (750 N). The black dots indicate the force measured by the sensor. The intermittent line represents the applied force by the bench press (750 N) with the 10% error range marked by dotted line. In the 20–37 °C range, the deviation was 1% at maximum. At extreme temperatures, the error fluctuates between +8% and −9%, but remains within the range of 10%; (**d**) Cyclic loading at 20 °C. Cyclic fluctuating forces between 70 N and 860 N, highlighted by the intermittent line, were applied to the sensor. The error between applied force and measured force was negligible (<1%).

Bench press testing has shown that the sensor accurately measured peak loads up to 1000 N with a sample frequency of 50 Hz in static situations at room temperature. A constant applied force on the sensor at increasing temperatures has shown that temperature changes had minimal effects on the stability and accuracy of the sensor between 20 °C to 37 °C. Cyclic bench press testing has shown that the sensor produced accurate results during cyclic loading for at least 500 s. Results of the static validation tests are illustrated in Figure 4. 

**Figure 5:**
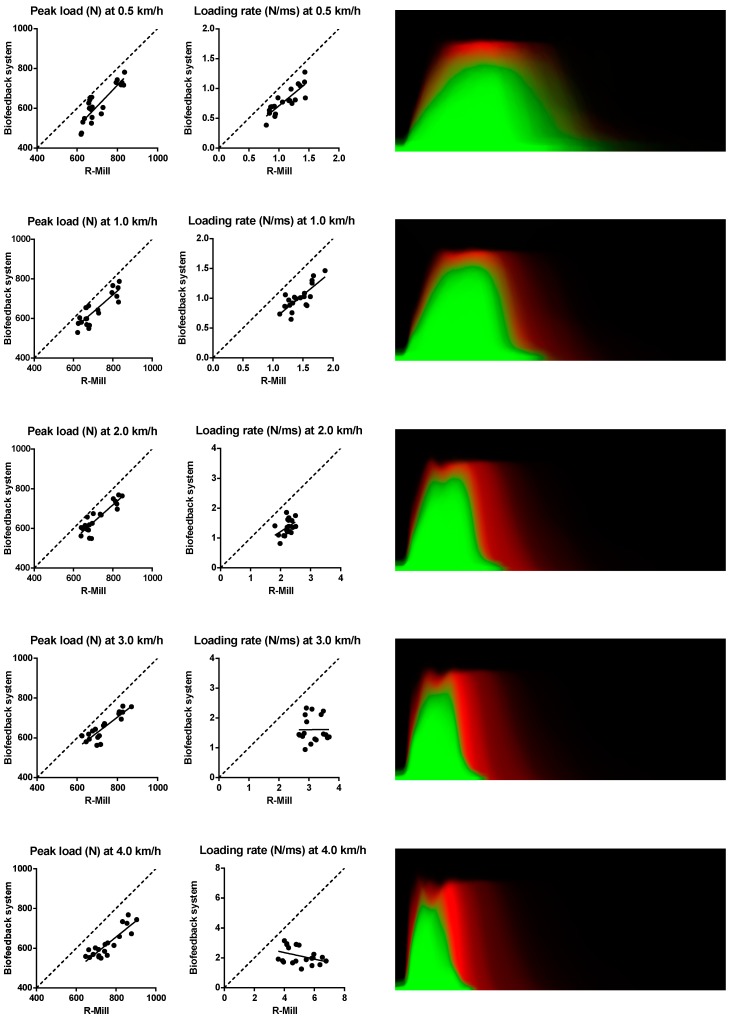
Validation of the biofeedback system in the dynamic situation at different speeds. **Left**: peak load (N) measured by the R-Mill vs. peak load (N) measured by the biofeedback system. Middle: loading rate (N/ms) measured by the R-Mill vs. loading rate (N/ms) measured by the biofeedback system. **Right**: overlay of the load curves (n = 10) measured by the R-Mill (red) and the biofeedback system (green). Legend: - - - Linear curve, 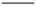 Observed curve, ● R-Mill vs. biofeedback system.

To validate the biofeedback system in the dynamic situation it was tested in 10 healthy volunteers (6 men and 4 women; mean age 24.9 years (SD 2.2 years); weighing 74 kg (SD 8.0 kg) and compared to a dual-belt instrumented treadmill: the R-Mill. The peak loads and loading rates at each speed measured in individual participants are presented in Figure 5. The peak loads measured by the biofeedback system were lower than the peak loads measured by the R-Mill. The same was observed in loading rates, where the difference between the two systems increased with speed. The differences in the two gait parameters are also represented by the load curves in Figure 5. It can be seen that the shape of the load curves were similar, but the biofeedback system curves were lower and less steep than the R-Mill curves. The relative and absolute errors are shown in Table 1 and Table 2.

## 4. Discussion

Measuring lower limb loading in the ambulatory situation has become a growing field of interest in recent decades. Various techniques have been developed, whereby the ambulatory, portable biofeedback systems seem to have the most potential for clinical implementation. Still, existing biofeedback systems suffer from limitations that prevent widespread use in healthcare. Our aim was to develop a new ambulatory biofeedback system that overcomes existing limitations and, in addition, is able to provide real-time feedback about lower limb loading to the patient and the healthcare provider. The development resulted in a biofeedback system that consists of advanced sensor technologies based on the Hall effect. Highly accurate results were obtained in the static situation. The results in the dynamic situation need to be improved in the ongoing development of the system. 

Walking is a highly dynamical procedure and therefore difficult to measure. Previous systems encounter problems such as sensor migration, creep, temperature and/or humidity drift and hysteresis [2,11]. These problems can be overcome by using a sensor based on the Hall effect, as correct detection of lower limb loading is ensured, independent of the exact loading point on the sensor. In addition, the sensor developed in the current study sends load measurements wireless and in real-time via BLE to a wrist device and tablet. This ensures a high level of mobility, prevents the use of cables, and has low power consumption. These are requirements for a user-friendly biofeedback system, that allows a comfortable, safe and natural gait of the user [1]. Moreover, the system enables the patient and healthcare provider to gain real-time insight in relevant load measurements and thereby the ability to adapt lower limb loading to the optimal level during training. 

In this study, the sensor was validated in a static situation and the entire biofeedback system, including the custom-made sandals, wrist device, and tablet, in the dynamic situation. The sensor was highly accurate in the static situation as shown by bench press testing. The sensor has a wide force range from 0 to 1000 N and shows accurate detection of loading in cyclic testing. Low temperature sensitivity has been observed between 20 °C and 37 °C, which is considered as the relevant range of daily usage [15]. 

Data generated by 10 healthy volunteers was compared to a dual-belt instrumented treadmill, as these systems are often used as the gold standard for gait analysis [10]. In this study, we found different results in gait parameters between the biofeedback system and the R-Mill. Important gait parameters as peak load and loading rate registered by the biofeedback system are lower than the data generated by the R-Mill at all speeds. Although it is known that gait patterns on a treadmill differ from overground walking [4], it is more likely that a suboptimal position of the sensor or the use of a single sensor results in lower gait parameters. From previous studies, it is known that plantar forces are different depending on the anatomic region under the foot. For example, peak force increases linearly with speed relative to the heel, lateral toe, and medial toe regions, while no significant differences were found with the midfoot, lateral forefoot, and central forefoot at different speeds [11]. In the experimental set-up of this study, the entire step is measured by the R-Mill, including the heelstrike and toestrike, while the biofeedback system only measured the midstance due to the sensor location under the midfoot region. Probably the plantar force is not entirely measured during heelstrike and toestrike, which lead to the lower gait parameters observed in this study. Possibly, to overcome this issue, multiple sensors covering all anatomic regions under the foot could be applied. Integration of data from multiple sensors, however, is certainly challenging and will result in a less user-friendly system. For these reasons, we have chosen to use one sensor in combination with the Hall effect. 

With the new biofeedback system previous limitations and problems have been minimized, such as the use of cables, sensor migration, and sensor placement. Results in the static situation as well as the shape of the load curves indicate that the sensor is highly accurate. However, some limitations are still present in this new biofeedback system. The gait parameters measured in the dynamic situation are lower than the gold standard. To overcome this, significant improvements should be made, for example, in the sensor position or in the use of multiple sensors. Improvements can also be made in the design of the custom-made sandals. Previous studies have shown that rigid shoes and insoles have a significant influence on gait parameters [16]. In the ongoing development of the biofeedback system, adjustments in the sandal design are envisaged to overcome, at least in part, these issues. A limitation in the study design is that the system was validated in the static situation by bench press testing and in the dynamic situation by use in healthy volunteers. Clinical application includes patients suffering from lower limb injuries, and data should ideally be validated in this group. Hypothetically, factors as pain and anxiety, lower walking speed and usage of crutches lead to lower results than observed in the healthy volunteers. 

## 5. Conclusions

An ambulatory biofeedback system for measuring lower limb loading has been developed that uses advanced sensor technologies. Real-time visual feedback about lower limb loading can be provided to the patient and the healthcare provider. Based on static measurements and the dynamic load curves, it can be concluded that the sensor is highly accurate. Measurements in the dynamic situation, however, indicate that sensor position and sandal design may be improved in further development. 

## Figures and Tables

**Table 1:** Average relative and absolute error in peak loads (n = 10).

Speed (km/h)	Relative Error (Min–Max)	Absolute Error in N (Min–Max)
0.5	−0.15 (−0.05 to −0.26)	−105.9 (−34.56 to −166.5)
1.0	−0.12 (−0.04 to −0.21)	−86.15 (−25.14 to −162.7)
2.0	−0.12 (−0.04 to −0.22)	−88.73 (−29.77 to −159.5)
3.0	−0.13 (−0.05 to −0.23)	−97.77 (−30.23 to −166.5)
4.0	−0.20 (−0.13 to −0.27)	−152.2 (−86.89 to −221.3)

**Table 2:** Average relative and absolute error in loading rates (n = 10).

Speed (km/h)	Relative Error (Min–Max)	Absolute Error in N/ms (Min–Max)
0.5	−0.29 (−0.11 to −0.51)	−0.03 (−0.01 to −0.06)
1.0	−0.31 (−0.12 to −0.50)	−0.04 (−0.02 to −0.07)
2.0	−0.38 (−0.15 to −0.59)	−0.09 (−0.03 to −0.12)
3.0	−0.48 (−0.20 to −0.67)	−0.16 (−0.06 to −0.24)
4.0	−0.57 (−0.21 to −1.27)	−0.30 (−0.09 to −0.51)
